# Chronic scrotal pain caused by Mild Epididymitis:Report of a series of 44 cases

**DOI:** 10.12669/pjms.303.4256

**Published:** 2014

**Authors:** Yongqing Lai, Zuhu Yu, Bentao Shi, Liangchao Ni, Yunchu Liu, Shangqi Yang

**Affiliations:** 1 Yongqing Lai, Department of Urology, Peking University Shenzhen Hospital, Shenzhen, 518036, P.R. China.; 2 Zuhu Yu, Department of Urology, Peking University Shenzhen Hospital, Shenzhen, 518036, P.R. China.; 3 Bentao Shi, Department of Urology, Peking University Shenzhen Hospital, Shenzhen, 518036, P.R. China.; 4 Liangchao Ni, Department of Urology, Peking University Shenzhen Hospital, Shenzhen, 518036, P.R. China.; 5 Yunchu Liu, Department of Urology, Peking University Shenzhen Hospital, Shenzhen, 518036, P.R. China.; 6 Shangqi Yang, Department of Urology, Peking University Shenzhen Hospital, Shenzhen, 518036, P.R. China.

**Keywords:** Chronic scrotal pain, Idiopathic, Mild Epididymitis, Underrecogniczed cause

## Abstract

***Objectives:*** Patients with idiopathic chronic scrotal pain are challenging to both the general practioner and urologist. In this study, we tried to recognize mild epididymitis as an underrecogniczed cause of idiopathic chronic scrotal pain.

***Methods*** *:* We described a consecutive series of 44 patients with idiopathic chronic scrotal pain characterized by mild scrotal pain, mild to moderate tenderness of epididymis without abnormal swelling of epididymis. We obtained a detailed history and physical examination along with routine urinalysis and Doppler ultrasound to identify the characteristics of this new clinical entity.

***Results*** *:* A consecutive series of 44 patients who were primarily diagnosed as "idiopathic chronic scrotal pain" came to our hospital. All had the sign of mild to moderate tenderness on the affected epididymis without epididymis enlargement. Doppler ultrasound showed the affected epididymis with normal size and no abnormal change. We treated them with antibiotics orally along with cessation of strenuous activity and all fully recovered from scrotal pain.

***Conclusion:*** In this study, we recognized mild epididymitis as an underrecogniczed cause of idiopathic chronic scrotal pain. It was characterized by mild scrotal pain, mild to moderate tenderness of epididymis without abnormal enlargement of epididymis.

## INTRODUCTION

In a general urologic practice, patients present frequently with chronic scrotal pain. Pain may be bothersome and without explanation, recurrent or persistent, and it may lead a patient to fear that he has a serious disease. In many cases scrotal pain is idiopathic. These patients are challenging to both the general practioner and urologist, and a clear understanding of the etiology and course of this common condition is needed when treating them.^1^

Epididymitis by definition is inflammation of the epididymis. This term of "mild epididymitis" is already fully known in the literature and reserved for specific forms of epididymitis.^2^^,^^3^ Desai et al^2^ classified epididymitis into 3 groups as mild, moderate and severe according to the degree of inflammation.

In this study, we tried to recognize mild epididymitis as an underrecogniczed cause of idiopathic chronic scrotal pain. This series of patients characterized by mild scrotal pain, mild to moderate tenderness of epididymis without abnormal enlargement of epididymis. For the reason of under-diagnosis and high incidence of mild epididymitis, we report a consecutive series of 44 cases of mild epididymitis.

## METHODS

We describe a consecutive series of 44 patients with idiopathic chronic scrotal pain characterized by mild scrotal pain, mild to moderate tenderness of epididymis without abnormal swelling of epididymis. Patients with potential causes of chronic scrotal pain^1^^,^^4^^,^^5^ were excluded. Patients with post-vasectomy, previous testicular trauma or surgery (including herniorrhaphy, hydrocele repair, and varicocelectomy), tumour, post-infection, post-torsion were excluded. We obtained a detailed history and physical examination along with routine urinalysis and Doppler ultrasound to identify the characteristics of this new clinical entity.

## RESULTS

From October 2011 to May 2012, we included a consecutive series of 44 patients who complained of mild scrotal pain over 6 weeks (Table-I) with mild to moderate tenderness of epididymis without abnormal enlargement of epididymis. They were primarily diagnosed as "idiopathic chronic scrotal pain".. All had the symptom of mild unilateral or bilateral scrotal pain, aggravation of pain after strenuous exercise and without acute onset of scrotal pain. All had the characteristic sign of mild to moderate tenderness on the affected epididymis without epididymis enlargement. Previously, all the 44 patients had no history of acute onset of scrotal pain and swelling of epididymis. None had positive routine urinalysis. The affected epididymis showed normal size and no abnormal change with Doppler ultrasound.

As shown in Table-I, the average age of this series was 33.8(20-49). The median duration of onset to first visit was 5 weeks (1-10 weeks). The median duration of onset to this visit was 43 weeks (6-102 weeks). The median duration of misdiagnosis was 36 weeks (4-98 weeks). 38/44 was affected unilateral involvement and 6/44 bilateral involvement.

We treated them with antibiotics(cephalosporins and quinolones are the first choice) orally along with cessation of strenuous activity. Whether the used antibiotics were effective depended on the relief of scrotal pain or tenderness of epididymis. If the Cephalosporins or Quinolones orally 1 week didn't work, we changed to Doxycycline and Azythromycin to continue the treatment. As shown in Table-I, the median duration of treatment was 4 weeks (2-8 weeks). The longer the duration of onset to this visit, the longer the duration of treatment. After the treatment, all the 44 patients fully recovered from scrotal pain and tenderness of epididymis.

**Table-I:** Characteristics of the 44 patients

*Patient No.*	*Age* *(yr)*	*Duration of * *onset* * to first visit (w)*	*Duration of * *onset* * to this visit (w)*	*Duration of misdiagnosis*	*Affected epididymis*	*Duration of treatment (w)*
1	38	1	6	5	U	2
2	22	5	21	16	B	4
3	36	10	14	4	U	4
4	26	10	102	92	U	7
5	39	7	42	35	U	5
6	48	8	73	65	U	5
7	21	7	31	24	U	4
8	35	3	30	27	B	4
9	20	5	14	9	U	3
10	34	7	85	78	U	8
11	45	4	94	90	U	8
12	34	8	20	12	U	2
13	35	9	57	48	U	5
14	37	10	46	36	U	4
15	46	2	65	63	U	8
16	35	8	55	47	U	7
17	32	9	102	93	U	8
18	24	2	75	73	B	6
19	20	7	30	23	U	4
20	28	7	64	57	U	4
21	42	6	84	78	B	7
22	40	6	64	58	U	8
23	38	7	87	80	U	6
24	20	6	22	16	U	4
25	29	4	77	73	U	6
26	41	5	16	11	U	3
27	46	5	33	28	U	3
28	42	6	43	37	U	3
29	33	10	42	32	U	4
30	29	3	100	97	B	8
31	39	5	67	62	U	6
32	48	1	19	18	U	2
33	21	4	90	86	U	8
34	31	10	39	29	U	4
35	49	1	19	18	U	3
36	29	3	101	98	U	8
37	33	3	96	93	U	7
38	43	4	67	63	U	6
39	29	10	33	23	U	4
40	22	2	36	34	B	4
41	20	4	27	23	U	4
42	35	9	50	41	U	6
43	48	3	19	16	U	4
44	29	1	58	57	U	6

U: Unilateral sides affected; B: Bilateral sides affected.

## DISCUSSION

Reports of chronic scrotal pain are obscured by differences in the selection of patients and in the definition of the condition.^6^ In published reports on this subject, the term chronic refers to periods varying from 1 week^7^ to 6 months.^8^ In this study, we defined chronic as lasting for at least 6 weeks.

Chronic scrotal pain poses a special challenge for the urologist. It is difficult to treat, as the cause is often unknown, and might require a multidisciplinary approach. In many patients, the clinical examination is often normal and investigations such as scrotal ultrasonography and microbiological urine analysis can be unremarkable.^4^ The pain is often severe enough to affect the daily activities of the patient, causing them to seek medical help.

There are many possible causes of chronic scrotal pain.^1^^,^^4^^,^^5^ Pain might be caused primarily by pathology in the scrotum or groin, or be referred from another area (‘referred pain’). Local causes can include chronic infection of the testis or epididymis, testicular tumour, indirect inguinal hernia, hydrocele, spermatocele or varicocele. Referred pain might originate from the ureter (ureteric stone), prostate (chronic prostatitis) or lumbar spine (degenerative lesions). However, in up to 25% of cases no identifiable cause for chronic scrotal pain is found, despite extensive investigation.^9^

Epididymitis by definition is inflammation of the epididymis. This term of "mild epididymitis" is already fully known in the literature and reserved for specific forms of epididymitis.^2^^,^^3^ Desai et al^2^ classified epididymitis into 3 groups as mild, moderate and severe according to the degree of inflammation and the clinical criteria of mild epididymitis is "thickened, tender epididymis testis separately palpable and non-tender". In this study, we found patients with mild epididymitis had no fever, no swelling of epididymis and just had mild scrotal pain, mild to moderate tenderness of epididymis. This and Desai 's study^2^ are basically the same.

Acute epididymitis represents sudden occurrence of pain and swelling of the epididymis associated with acute inflammation of the epididymis.^10^ Chronic epididymitis refers to inflammation and pain in the epididymis persisting for over 6 weeks.^10^ As shown in Table-I, 22/44 visit doctor over 6 after the onset. That may be due the relatively mild symptoms and signs. Unfortunately, 22/44 seek medical treatment in 6 weeks after the onset and no patients got the right treatment in our department in 6 weeks after the onset. The duration of misdiagnosis was too long (median duration: 36 weeks) . Therefore, it is vital to recognize this clinical entity to resolve the pain of this group of patients.

A consecutive series of 44 patients were enrolled in our study. They didn't seek medical treatment immediately (the median duration of onset to first visit was 5 weeks). Most (38/44) were unilateral involvement. We tried to treat them with antibiotics orally along with cessation of Strenuous activity. A 1- to 8-week trial of antibiotics should potentially be effective against possible bacterial pathogens. As shown in Table-I, the longer the duration of onset for the first visit, the longer the duration of treatment. Therefore, it is very important for urologists to recognize and treat this clinical entity early.

In many patients with chronic scrotal pain, no clinical abnormalities can be found. There is a tendency not to rely any longer on physical examination alone^6^. When no diagnosis can be made on the basis of a thorough physical examination, a scrotal ultrasound is often performed.^6^ Although several studies have demonstrated the accuracy of diagnostic ultrasound of the scrotum and its contents, in patients with chronic scrotal pain and a normal physical examination, there is no scientific basis to perform this test.^6^ In this study, we have shown that all the patients with mild epididymitis had normal epididymis results of ultrasound. Its characteristic sign is mild to moderate tenderness of epididymis without abnormal enlargement of epididymis. After checking carefully, we can easily determine the diagnosis. Doctors should learn again to put their trust in their abilities to perform a physical examination of the scrotum.^6^ Although there is little harm, other than economic reasons and its burden on radiologic services, to use a simple and fast modality such as ultrasound, its main indication seems to be to reassure the patient and his doctor that no serious pathologic features are present.^6^

## CONCLUSION

In this study, we have recognized mild epididymitis as an underrecogniczed cause of idiopathic chronic scrotal pain. This series of patients was characterized by mild scrotal pain, mild to moderate tenderness of epididymis without abnormal enlargement of epididymis. After the treatment, all the 44 patients recovered from the scrotal pain. 
